# Supplementary material for: Novel Technique to Measure Pulse Wave Velocity in Brain Vessels Using a Fast Simultaneous Multi-Slice Excitation Magnetic Resonance Sequence
Source: Sensors (Basel). 2021 Sep 23;21(19):6352. doi: 10.3390/s21196352 (PMC8512313; doi:10.3390/s21196352)
Supplement: Supplementary file 1 [file sensors-21-06352-s001.zip › sensors-1369551-supplementary.pdf]

Supplementary Materials: Novel Technique to Measure Pulse Wave Velocity in Brain Vessels Using a Fast Simultaneous Multi-Slice Excitation Magnetic Resonance Sequence

Ju-Yeon Jung [1], Yeong-Bae Lee [2,3,*] and Chang-Ki Kang [1,2,4,*]

1 Department of Health Science, Gachon University Graduate School, Gachon University, Incheon 21936, Korea; 9955me@gachon.ac.kr
2 Neuroscience Research Institute, Gachon University, Incheon 21565, Korea
3 Department of Neurology, Gil Medical Center, Gachon University College of Medicine, Incheon 21565, Korea
4 Department of Radiological Science, College of Health Science, Gachon University, Incheon 21936, Korea
* Correspondence: yeongbaelee@gachon.ac.kr (Y.-B.L.); ckkang@gachon.ac.kr (C.-K.K.); Tel.: +82-32-460-3346 (Y.-B.L.); +82-32-820-4110 (C.-K.K.)

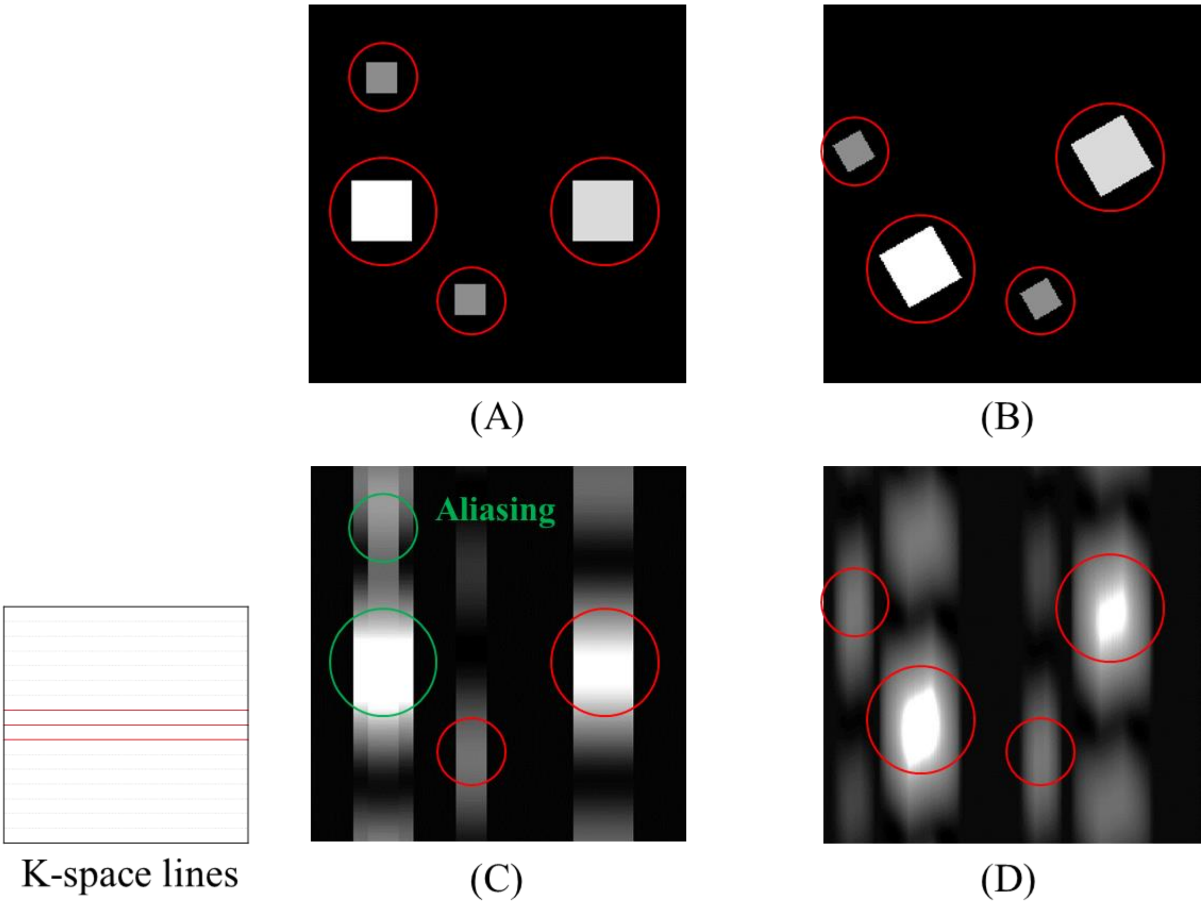

Figure S1. Simulation results of SMS-K technique for avoiding aliasing artifacts. (A) Original image which can cause the aliasing artifact in SMS-K technique. (B) Rotated image which is rotated 30 degrees counterclockwise in image A. (C and D) Reconstructed images of (A) and (B), respectively. Note that only 3 k-space lines were used for image reconstruction. Circles indicate the locations of the simulated structures, and the green ones produce aliased signals.

.
